# Supplementary material for: Rapid Screening of Microalgae as Potential Sources of Natural Antioxidants
Source: Foods. 2023 Jul 10;12(14):2652. doi: 10.3390/foods12142652 (PMC10378671; doi:10.3390/foods12142652)
Supplement: Supplementary file 1 [file foods-12-02652-s001.zip › foods-2465698-supplementary.pdf]

Rapid Screening of Microalgae as Potential Sources of Natural Antioxidants

Na Wang [1,2,†], Haiwei Pei [1,2,†], Wenzhou Xiang [1,3], Tao Li [1,3], Shengjie Lin [4], Jiayi Wu [1,3], Zishuo Chen [1,2], Houbo Wu [1,3], Chuanmao Li [4], Hualian Wu [1,3,*]

1 CAS Key Laboratory of Tropical Marine Bio-resources and Ecology, Guangdong Key Laboratory of Marine Materia Medica, RNAM Center for Marine Microbiology, Institution of South China Sea Ecology and Environmental Engineering, South China Sea Institute of Oceanology, Chinese Academy of Sciences, Guang-zhou 510301, China; nawang@scsio.ac.cn (N.W.); haiweipei@scsio.ac.cn (H.P.); xwz@scsio.ac.cn (W.X.); taoli@scsio.ac.cn (T.L.); kayeewu@scsio.ac.cn (J.W.); 18390943716@163.com (Z.C.); wuhoubo@scsio.ac.cn (H.W.)

2 University of Chinese Academy of Sciences, Beijing 100049, China

3 Southern Marine Science and Engineering Guangdong Laboratory (Guangzhou), No. 1119, Haibin Road, Nansha District, Guangzhou 511458, China

4 Guangzhou Keneng Cosmetic Scientific Research Co. Ltd, Guanghzou 510000, China; linsj@danzi.cn (S.L.); lichm@danzi.cn (C.L.)

* Correspondence: hlwu@scsio.ac.cn (H.W.); Tel.: +86-20-89023195 (H.W.)

† These authors contributed equally to this work

Submitted to *Foods*

Supplementary tables

Table S1

Table S1. Information of 16 microalgae species

Serial number	Species	Classes	Medium	Source
SCSIO-46784	*Chlorella sorokiniana*	Chlorophyta	BG-11	Economic Microalgae Culture Collection of Guandong Province (South China Sea Institute of Oceanography, Chinese Academy of Sciences)
SCSIO-46781	*Euglena gracilis*	Euglenophyta	EGM	Freshwater Algae Culture of Hydrobiology Collection at the Institute of Hydrobiology, Chinese Academy of Sciences (Wuhan, China), Strain No. FACHB-849
SCSIO-46716	*Eustigmatos* sp.	Chlorophyta	BG-11	Economic Microalgae Culture Collection of Guandong Province (South China Sea Institute of Oceanography, Chinese Academy of Sciences)
SCSIO-45217	*Nannochloropsis* sp.	Chrysophyta	F/2	Economic Microalgae Culture Collection of Guandong Province (South China Sea Institute of Oceanography, Chinese Academy of Sciences)
SCSIO-45006	*Nannochloropsis* sp.	Chrysophyta	F/2	Economic Microalgae Culture Collection of Guandong Province (South China Sea Institute of Oceanography, Chinese Academy of Sciences)
SCSIO-45224	*Nannochloropsis* sp.	Chrysophyta	F/2	Economic Microalgae Culture Collection of Guandong Province (South China Sea Institute of Oceanography, Chinese Academy of Sciences)
SCSIO-45120	*Phaeodactylum tricornutum*	Becillariophyta	F/2	Economic Microalgae Culture Collection of Guandong Province (South China Sea Institute of Oceanography, Chinese Academy of Sciences)
SCSIO-45949	*Porphyridium cruentum*	Rhodophyta	ASW	Economic Microalgae Culture Collection of Guandong Province (South China Sea Institute of Oceanography, Chinese Academy of Sciences)
SCSIO-45707	*Rhodosorus* sp	Rhodophyta	ASW	Economic Microalgae Culture Collection of Guandong Province (South China Sea

				Institute of Oceanography, Chinese Academy of Sciences)
SCSIO-46548	*Asterarcys* sp.	Chlorophyta	ZSNT (fresh water)	Economic Microalgae Culture Collection of Guandong Province (South China Sea Institute of Oceanography, Chinese Academy of Sciences)
SCSIO-45829	*Asterarcys* sp.	Chlorophyta	ZSNT (seawater)	Economic Microalgae Culture Collection of Guandong Province (South China Sea Institute of Oceanography, Chinese Academy of Sciences)
SCSIO-46585	*Scenedesmus* sp.	Chlorophyta	BG-11	Economic Microalgae Culture Collection of Guandong Province (South China Sea Institute of Oceanography, Chinese Academy of Sciences)
SCSIO-46579	*Scenedesmus* sp.	Chlorophyta	BG-11	Economic Microalgae Culture Collection of Guandong Province (South China Sea Institute of Oceanography, Chinese Academy of Sciences)
SCSIO-46591	*Scenedesmus* sp.	Chlorophyta	BG-11	Economic Microalgae Culture Collection of Guandong Province (South China Sea Institute of Oceanography, Chinese Academy of Sciences)
SCSIO-44012	*Arthrospira platensis*	Cyanophyta	Zarrouk	Economic Microalgae Culture Collection of Guandong Province (South China Sea Institute of Oceanography, Chinese Academy of Sciences)
SCSIO-46782	*Uronema* sp.	Chlorophyta	BG-11	Economic Microalgae Culture Collection of Guandong Province (South China Sea Institute of Oceanography, Chinese Academy of Sciences)

Table S2

Table S2. Components of BG11 medium

Serial number	Compound	Concentration
1	$NaNO_3$	1.5 g L^{-1}
2	$Na_2HPO_4·3H_2O$	40 mg L^{-1}
3	$MgSO_4·7H_2O$	75 mg L^{-1}
4	$CaCl_2·2H_2O$	36 mg L^{-1}
5	$C_6H_8FeNO_7$	6 mg L^{-1}
6	$C_6H_8O_7$	6 mg L^{-1}
7	EDTA	1 mg L^{-1}
8	Na_2CO_3	20 mg L^{-1}
9	A_5	1 mL L^{-1}

Table S3

Table S3. Components of A_5

Serial number	Compound	Concentration
1	H_3BO_3	2.86 g L^{-1}
2	$MnCl_2 \cdot 4H_2O$	1.81 g L^{-1}
3	$ZnSO_4 \cdot 7H_2O$	0.22 g L^{-1}
4	$Na_2MoO_4 \cdot 2H_2O$	0.39 g L^{-1}
5	$CuSO_4 \cdot 5H_2O$	0.08 g L^{-1}
6	$Co(NO_3)_2 \cdot 6H_2O$	0.05 g L^{-1}

Table S4

Table S4. Components of F/2 medium

Serial number	Compound	Concentration
1	$NaNO_3$	75 mg L^{-1}
2	$NaH_2PO_4 \cdot 2H_2O$	5 mg L^{-1}
3	$Na_2SiO_3 \cdot 9H_2O$	30 mg L^{-1}
4	F/2 trace metal solution	1 mL L^{-1}
5	F/2 vitamin solution	0.5 mL L^{-1}

Table S5

Table S5. Components of F/2 trace metal solution

Serial number	Compound	Concentration
1	$FeCl_3{\cdot}6H_2O$	3.15 g L^{-1}
2	$EDTANa_2{\cdot}2H_2O$	4.36 g L^{-1}
3	$CuSO_4{\cdot}5H_2O$	9.8 mg L^{-1}
4	$Na_2MoO_4{\cdot}2H_2O$	6.3 mg L^{-1}
5	$ZnSO_4{\cdot}7H_2O$	22 mg L^{-1}
6	$CoCl_2{\cdot}6H_2O$	10 mg L^{-1}
7	$MnCl_2{\cdot}4H_2O$	180 mg L^{-1}

Table S6

Table S6. Components of F/2 vitamin solution

Serial number	Compound	Concentration
1	Vitamin B_{12}	1 mg L^{-1}
2	Biotin	1 mg L^{-1}
3	Thiamine·HCl	0.2 g L^{-1}

Table S7

Table S7. Components of ASW medium

Serial number	Compound	Concentration
1	$NaCl$	27 g L^{-1}
2	$MgSO_4{\cdot}7H_2O$	6.6 g L^{-1}
3	$MgCl_2{\cdot}6H_2O$	5.6 g L^{-1}
4	$CaCl_2{\cdot}2H_2O$	1.5 g L^{-1}
5	KNO_3	1.45 g L^{-1}
6	K_2HPO_4	0.12 g L^{-1}
7	$EDTANa_2{\cdot}2H_2O$	4.36 mg L^{-1}
8	$FeCl_3{\cdot}6H_2O$	3.15 mg L^{-1}
9	$MnCl_2{\cdot}4H_2O$	180 μg L^{-1}
10	$ZnSO_4{\cdot}7H_2O$	22 μg L^{-1}
11	$Na_2MoO_4{\cdot}2H_2O$	6 μg L^{-1}
12	$CoCl_2{\cdot}6H_2O$	10 μg L^{-1}
13	$CuSO_4{\cdot}5H_2O$	10 μg L^{-1}

Table S8

Table S8. Components of modified ZSNT medium

Serial number	Compound	Concentration
1	$NaNO_3$	1.5 g L^{-1}
2	$NaH_2PO_4·2H_2O$	0.05 g L^{-1}
3	$CaCl_2$	0.02 g L^{-1}
4	$MgSO_4·7H_2O$	0.05 g L^{-1}
5	KCl	0.1 g L^{-1}
6	K_2SO_4	0.3 g L^{-1}
7	M_2	1 mL L^{-1}
8	A_5	1 mL L^{-1}

Table S9

Table S9. Components of M_2

Serial number	Compound	Concentration
1	$FeCl_3 \cdot 6H_2O$	2.44 g L^{-1}
2	$EDTANa_2 \cdot 2H_2O$	1.89 g L^{-1}

Table S10

Table S10. Components of modified EGM medium

Serial number	Compound	Concentration
1	NH_4Cl	0.83 g L^{-1}
2	KH_2PO_4	1 g L^{-1}
3	$MgSO_4{\cdot}7H_2O$	0.2 g L^{-1}
4	$CaCl_2$	0.02 g L^{-1}
5	$Fe_2(SO_4)_3$	3 mg L^{-1}
6	$EDTANa_2{\cdot}2H_2O$	0.48 mg L^{-1}
7	Vitamin B_1	0.1 mg L^{-1}
8	Vitamin B_{12}	0.1 μg L^{-1}
9	A_5	1 mL L^{-1}

Table S11

Table S11. Components of Zarrouk medium

Serial number	Compound	Concentration
1	$NaHCO_3$	5 g L^{-1}
2	$NaNO_3$	0.5 g L^{-1}
3	$FeSO_4{\cdot}7H_2O$	5 mg L^{-1}
4	$NaH_2PO_4{\cdot}2H_2O$	0.02 g L^{-1}
5	K_2SO_4	0.5 g L^{-1}
6	$MgSO_4{\cdot}7H_2O$	0.5 g L^{-1}
7	$CaCl_2$	0.04 g L^{-1}
8	A_5	1 mL L^{-1}

Table S12

Table S12. Carotenoids contents (mg g^{-1} biomass) of the extracts from 16 microalgae strains

Species	One step	Three steps			
	ethanol/water	hexane	ethylacetate	water	total
Chlorella sorokiniana SCSIO-46784	1.02±0.04[b]	0.28±0.01	0.23±0.01	N.D.	0.51±0.02[a]
Euglena gracilis SCSIO-46781	3.71±0.14[a]	2.23±0.02	3.84±0.10	N.D.	6.07±0.12[b]
Eustigmatos sp. SCSIO-46716	0.08±0.00[a]	0.05±0.00	0.03±0.00	0.01±0.00	0.09±0.01[b]
Nannochloropsis sp. SCSIO-45217	0.68±0.00[b]	0.21±0.03	0.22±0.01	0.14±0.00	0.57±0.03[a]
Nannochloropsis sp. SCSIO-45006	0.79±0.05[a]	0.40±0.02	0.47±0.00	N.D.	0.86±0.02[a]
Nannochloropsis sp. SCSIO-45224	2.37±0.05[a]	0.86±0.01	1.40±0.03	0.41±0.01	2.67±0.02[b]
Phaeodactylum tricornutum SCSIO-45120	5.45±0.09[b]	0.20±0.00	0.28±0.01	0.10±0.00	0.58±0.01[a]
Porphyridium cruentum SCSIO-45949	0.37±0.03[a]	0.37±0.03	N.D.	0.04±0.04	0.41±0.07[a]
Rhodosorus sp. SCSIO-45707	0.02±0.00[a]	0.01±0.00	0.01±0.00	0.03±0.00	0.05±0.01[b]
Asterarcys sp. SCSIO-46548	1.14±0.02[a]	0.44±0.04	1.13±0.06	0.20±0.03	1.77±0.13[b]
Asterarcys sp. SCSIO-45829	2.06±0.05[a]	1.17±0.14	2.37±0.20	0.17±0.01	3.61±0.35[b]
Scenedesmus sp. SCSIO-46585	0.29±0.00[b]	0.02±0.00	0.03±0.00	N.D.	0.05±0.00[a]
Scenedesmus sp. SCSIO-46579	0.86±0.06[b]	0.04±0.01	0.05±0.00	N.D.	0.10±0.01[a]
Scenedesmus sp. SCSIO-46591	0.10±0.00[b]	0.02±0.00	0.04±0.00	N.D.	0.06±0.01[a]
Arthrospira platensis SCSIO-44012	1.36±0.06[a]	1.03±0.03	0.32±0.01	0.18±0.02	1.53±0.01[a]
Uronema sp. SCSIO-46782	2.22±0.03[b]	0.16±0.01	0.13±0.01	0.01±0.01	0.31±0.01[a]

Note: N.D. = not detectable; different letters indicated significant differences in carotenoids contents of microalgae extracts obtained by one-step and three-step methods (p<0.05).

Table S13

Table S13. Phenols contents (mg g^{-1} biomass) of the extracts from 16 microalgae strains

Species	One step	Three steps			
	ethanol/water	hexane	ethylacetate	water	total
Chlorella sorokiniana SCSIO-46784	1.81±0.09[a]	1.08±0.05	N.D.	1.07±0.05	2.16±0.04[b]
Euglena gracilis SCSIO-46781	3.90±0.06[a]	4.31±0.07	6.26±0.25	1.16±0.03	11.73±0.22[b]
Eustigmatos sp. SCSIO-46716	0.58±0.01[a]	0.37±0.02	0.28±0.00	1.23±0.05	1.88±0.06[b]
Nannochloropsis sp. SCSIO-45217	1.23±0.01[a]	1.34±0.09	0.53±0.04	0.50±0.03	2.36±0.15[b]
Nannochloropsis sp. SCSIO-45006	2.80±0.30[a]	2.55±0.43	1.19±0.04	1.39±0.12	5.13±0.58[b]
Nannochloropsis sp. SCSIO-45224	2.39±0.06[a]	1.51±0.06	2.07±0.20	1.88±0.05	5.46±0.23[b]
Phaeodactylum tricornutum SCSIO-45120	3.69±0.03[b]	0.32±0.02	0.40±0.03	1.77±0.01	2.50±0.02[a]
Porphyridium cruentum SCSIO-45949	0.94±0.02[a]	0.49±0.03	0.89±0.01	0.61±0.10	1.99±0.11[b]
Rhodosorus sp. SCSIO-45707	0.43±0.05[a]	0.23±0.01	0.25±0.01	0.38±0.01	0.86±0.02[b]
Asterarcys sp. SCSIO-46548	2.90±0.23[b]	0.23±0.05	0.18±0.03	1.90±0.28	2.31±0.36[a]
Asterarcys sp. SCSIO-45829	2.89±0.06[a]	0.62±0.36	0.13±0.05	3.01±0.06	3.76±0.47[b]
Scenedesmus sp. SCSIO-46585	1.51±0.02[a]	0.30±0.03	N.D.	1.45±0.03	1.76±0.05[b]
Scenedesmus sp. SCSIO-46579	1.93±0.24[a]	0.24±0.02	0.29±0.02	1.03±0.12	1.56±0.11[a]
Scenedesmus sp. SCSIO-46591	1.15±0.04[a]	0.21±0.02	0.22±0.01	1.23±0.07	1.66±0.08[b]
Arthrospira platensis SCSIO-44012	1.45±0.12[a]	0.82±0.05	2.59±0.12	2.03±0.06	5.44±0.11[b]
Uronema sp. SCSIO-46782	2.17±0.00[a]	0.39±0.03	0.77±0.15	1.05±0.12	2.21±0.03[a]

Note: N.D. = not detectable; different letters indicated significant differences in phenols contents of microalgae extracts obtained by one-step and three-step methods ($p<0.05$).

Table S14

Table S14. ABTS radicals scavenging capacity of the ethanol/water extraction extracts and the fractionating procedure extracts from 16 microalgae strains

Species	One step	Three steps			
	ethanol/water	hexane	ethylacetate	water	total
Chlorella sorokiniana SCSIO-46784	24.55±0.97[a]	6.74±0.15	7.98±0.37	12.76±1.19	27.49±1.06[a]
Euglena gracilis SCSIO-46781	29.60±0.95[a]	23.13±2.16	19.52±1.42	13.40±0.37	56.06±3.45[b]
Eustigmatos sp. SCSIO-46716	6.82±0.37[a]	1.94±0.04	2.00±0.10	7.80±0.13	11.73±0.20[b]
Nannochloropsis sp. SCSIO-45217	8.95±0.70[a]	5.52±0.53	4.84±1.73	4.87±0.38	15.23±1.68[b]
Nannochloropsis sp. SCSIO-45006	18.22±0.21[a]	6.11±0.16	7.93±0.28	14.29±0.56	28.33±0.56[b]
Nannochloropsis sp. SCSIO-45224	28.80±0.27[b]	7.63±0.39	8.01±0.14	8.69±0.42	24.33±0.68[a]
Phaeodactylum tricornutum SCSIO-45120	22.62±0.05[a]	2.09±0.15	2.96±0.12	22.10±0.30	27.15±0.25[b]
Porphyridium cruentum SCSIO-45949	5.80±0.65[a]	4.70±0.16	5.77±0.39	5.57±0.38	16.05±0.72[b]
Rhodosorus sp. SCSIO-45707	3.67±0.09[a]	1.19±0.32	1.54±0.15	5.63±0.03	8.36±0.43[b]
Asterarcys sp. SCSIO-46548	17.12±0.16[b]	N.D.	N.D.	5.86±0.47	5.86±0.47[a]
Asterarcys sp. SCSIO-45829	20.32±0.12[b]	1.47±0.33	0.09±0.02	8.11±1.07	9.67±1.42[a]
Scenedesmus sp. SCSIO-46585	13.63±0.62[a]	N.D.	N.D.	5.55±0.13	12.44±0.34[a]
Scenedesmus sp. SCSIO-46579	15.21±1.01[b]	N.D.	0.29±0.12	6.01±0.62	6.30±0.51[a]
Scenedesmus sp. SCSIO-46591	8.85±0.70[a]	N.D.	N.D.	7.12±0.77	7.12±0.77[a]
Arthrospira platensis SCSIO-44012	20.97±1.15[a]	6.03±0.35	14.98±0.57	21.72±0.05	42.73±0.53[b]
Uronema sp. SCSIO-46782	24.72±0.25[a]	3.50±0.07	4.56±0.25	14.04±0.86	22.10±1.12[a]

Note: N.D. = not detectable; different letters indicated significant differences in ABTS radicals scavenging capacity of microalgae extracts obtained by one-step and three-step methods (p<0.05).

Table S15

Table S15. DPPH radicals scavenging capacity of the ethanol/water extraction extracts and the fractionating procedure extracts from 16 microalgae strains

Species	One step	Three steps			
	ethanol/water	hexane	ethylacetate	water	total
Chlorella sorokiniana SCSIO-46784	9.99±0.62[a]	3.94±0.41	2.37±0.04	3.00±0.39	9.31±0.58[a]
Euglena gracilis SCSIO-46781	16.84±0.38[a]	15.49±0.69	13.43±0.74	5.12±0.41	34.04±1.66[b]
Eustigmatos sp. SCSIO-46716	5.26±0.19[a]	1.31±0.51	0.10±0.08	2.57±0.14	3.97±0.63[a]
Nannochloropsis sp. SCSIO-45217	5.43±0.13[a]	7.83±0.25	2.71±0.15	2.05±0.28	12.60±0.52[b]
Nannochloropsis sp. SCSIO-45006	7.89±0.28[a]	7.31±0.63	4.87±0.68	7.60±0.34	19.78±0.41[b]
Nannochloropsis sp. SCSIO-45224	14.20±0.18[a]	4.11±0.56	7.26±0.10	7.99±0.56	19.36±0.10[b]
Phaeodactylum tricornutum SCSIO-45120	18.27±0.69[b]	1.60±0.17	3.88±0.10	3.20±0.14	8.69±0.41[a]
Porphyridium cruentum SCSIO-45949	2.58±0.04[a]	2.48±0.10	2.72±0.13	3.25±0.30	8.45±0.45[b]
Rhodosorus sp. SCSIO-45707	2.45±0.48[a]	N.D.	N.D.	1.46±0.11	1.46±0.11[a]
Asterarcys sp. SCSIO-46548	10.14±0.65[a]	0.93±0.13	3.02±0.02	4.88±0.11	8.83±0.26[a]
Asterarcys sp. SCSIO-45829	10.26±0.46[a]	2.60±0.19	4.22±0.02	2.79±0.00	9.61±0.21[a]
Scenedesmus sp. SCSIO-46585	7.23±0.08[b]	N.D.	1.84±0.13	3.83±0.29	5.67±0.30[a]
Scenedesmus sp. SCSIO-46579	9.81±0.22[b]	N.D.	0.46±0.06	2.30±0.08	2.76±0.13[a]
Scenedesmus sp. SCSIO-46591	2.06±0.18[a]	1.97±0.97	0.71±0.21	2.43±0.83	5.12±1.79[a]
Arthrospira platensis SCSIO-44012	19.99±1.13[a]	5.94±1.06	12.03±1.14	3.45±0.30	21.42±2.24[a]
Uronema sp. SCSIO-46782	12.48±0.23[b]	2.17±0.08	2.26±0.13	3.71±0.54	8.14±0.69[a]

Note: N.D. = not detectable; different letters indicated significant differences in DPPH radicals scavenging capacity of microalgae extracts obtained by one-step and three-step methods ($p<0.05$).

Table S16

Table S16. FRAP activity of the ethanol/water extraction extracts and the fractionating procedure extracts from 16 microalgae strains

Species	One step	Three steps			
	ethanol/water	hexane	ethylacetate	water	total
Chlorella sorokiniana SCSIO-46784	22.90±0.39[a]	9.98±0.67	9.27±0.66	4.54±0.12	23.80±0.43[a]
Euglena gracilis SCSIO-46781	56.49±1.43[a]	44.71±4.18	83.79±1.89	4.12±1.12	132.62±7.01[b]
Eustigmatos sp. SCSIO-46716	3.69±0.33[a]	1.83±0.15	1.73±0.15	3.17±0.66	6.73±0.45[b]
Nannochloropsis sp. SCSIO-45217	16.33±1.45[a]	5.10±0.40	4.40±0.17	5.36±0.23	14.86±0.60[a]
Nannochloropsis sp. SCSIO-45006	13.20±0.57[a]	6.79±1.22	6.19±0.26	5.30±0.15	18.28±1.05[b]
Nannochloropsis sp. SCSIO-45224	30.89±0.68[a]	7.70±0.42	16.94±0.04	11.37±0.40	36.01±0.77[b]
Phaeodactylum tricornutum SCSIO-45120	45.42±2.28[b]	4.08±0.51	6.05±0.20	9.33±0.98	19.46±1.37[a]
Porphyridium cruentum SCSIO-45949	10.80±0.35[a]	4.76±0.30	6.88±0.21	2.39±0.09	14.03±0.10[b]
Rhodosorus sp. SCSIO-45707	4.66±0.67[a]	3.02±1.12	1.58±0.85	3.74±0.25	8.34±1.85[a]
Asterarcys sp. SCSIO-46548	28.02±1.85[b]	1.10±0.08	0.75±0.02	15.21±0.77	17.06±0.87[a]
Asterarcys sp. SCSIO-45829	35.42±0.54[b]	1.37±0.31	N.D.	10.02±0.26	11.39±0.57[a]
Scenedesmus sp. SCSIO-46585	11.43±0.26[b]	0.34±0.05	2.30±0.68	5.86±0.25	8.50±0.46[a]
Scenedesmus sp. SCSIO-46579	30.45±1.40[b]	1.36±0.07	0.97±0.04	5.15±0.04	7.48±0.08[a]
Scenedesmus sp. SCSIO-46591	11.49±0.60[a]	1.39±0.04	1.34±0.11	4.05±0.18	6.78±0.30[a]
Arthrospira platensis SCSIO-44012	17.50±0.94[a]	7.48±0.52	22.96±1.98	20.66±0.67	51.10±2.59[b]
Uronema sp. SCSIO-46782	19.47±1.03[a]	5.06±0.74	4.12±0.63	9.06±1.52	18.24±1.09[a]

Note: N.D. = not detectable; different letters indicated significant differences in FRAP activity of microalgae extracts obtained by one-step and three-step methods ($p<0.05$).
